# Metastatic NSCLC patients in the real world in Finland

**DOI:** 10.2340/1651-226X.2025.44254

**Published:** 2025-12-15

**Authors:** Heikki Ekroos, Olivia Hölsä, Anna Kreutzman, Lila Nikkola, Johanna Vikkula, Riikka Mattila, Aija Knuuttila

**Affiliations:** a HUS, Helsinki University Hospital, Helsinki, Finland; b Medaffcon Oy, Espoo, Finland; c Bristol Myers Squibb, Helsinki, Finland

**Keywords:** Carcinoma, non-small-cell lung, retrospective studies, neoplasm metastasis, immune checkpoint inhibitors, treatment outcome

## Abstract

**Background:**

Significant progress has been made in the management of metastatic NSCLC (mNSCLC). Our study investigated characteristics, treatment patterns, and outcomes in this patient population and subgroups based on histology and PD-L1 status.

**Methods:**

We conducted a retrospective analysis of electronic health records of patients with mNSCLC 1/2019-8/2023 at HUS, Helsinki University Hospital, Finland. Patient characteristics, treatment, and outcomes were analyzed.

**Results:**

We identified 646 patients with mNSCLC, including those metastatic at diagnosis and whose cancer later progressed to metastatic, who received systemic therapy. Median age was 70 years (interquartile range [IQR]: 62–75). Squamous cell carcinoma (SqC) presented 19% of patients, adenocarcinoma 68%, and other non-SqC 13%. Amongst the non-SqC patients 53% were female, whereas only 32% of SqC patients were female. Treatment evolved considerably, with increased use of immuno-oncology (IO) and tyrosine kinase inhibitor (TKI) therapies. Median overall survival was 8 months (confidence interval [CI] 95%: 6–9) for those treated with chemotherapy alone, 12 months (CI 95%: 7–18) for those treated with IO therapy, 14 months (CI 95%: 11–15) for those treated with IO + chemotherapy, and 24 months (CI 95%: 16–38) for those treated with TKIs.

**Interpretation:**

Our study reports real-world management of patients with mNSCLC and evolving treatment patterns in clinical practice from the first years of IO treatment availability. As we continue to monitor more recent data, the proportion receiving chemotherapy alone is anticipated to continue decreasing. It is crucial to assess current outcomes in NSCLC to target resources correctly and improve prognosis.

## Introduction

Approximately 3,000 new cases of lung cancer are diagnosed annually in Finland, with approximately 25% of cases occurring in the Helsinki University Hospital (HUS) area, making it the third most frequently diagnosed malignancy [1]. The incidence of lung cancer in Finland has remained lower than in Denmark and Norway over recent decades, though temporal trends differ across countries [2]. Survival rates following a lung cancer diagnosis remain poor with a 1-year relative survival of 42.9% [1]. However, high-quality data describing real-world outcomes for this patient group are scarce in many Western countries, highlighting the need for further research.

Non-small cell lung cancer (NSCLC) is the most common type of lung cancer, accounting for approximately 80% of all lung cancer diagnoses [1, 3]. NSCLC itself is a heterogenous group of subtypes, with adenocarcinoma (45%) and SqC (24%) being the most prevalent. Managing NSCLC is often challenging due to its aggressiveness, early propensity for metastases [4], and resistance to common therapies [5].

NSCLC is at the forefront of personalized medicine with genetic tests and targeted medications in routine use. Genetic testing is routinely performed for non-SqC patients and non-smoking SqC patients. The field is developing with new research findings, new genetic factors, and new targeted drugs, as well as immuno-oncology therapies coming to the market. This is expected to lead to better outcomes for the patients, and also to a very complex, and continually evolving treatment landscape.

Real-world data collected along routine patient care are needed to assess patient populations outside the context of randomized clinical trials and to extend the study population for the assessment of rare events and objectives. In addition, the ever-changing treatment landscape of lung cancer increases the need for a topical real-world evidence (RWE) study. Finland lacks a lung cancer registry, and access to up-to-date real-world data with deep clinical insight into, for example, treatment and metastasis is possible only through hospital data lakes that do not cover the whole country. Some RWE studies on lung cancer patients and their outcomes in Finland have been published [6–12], but mostly information on Finnish metastatic NSCLC (mNSCLC) patients is lacking.

In this retrospective data lake study, we investigated the real-world management of mNSCLC patients in the HUS area. This region, which provides universal healthcare, covers 1.7 million inhabitants, approximately 30% of Finnish population. The aim of this RWE study was to understand the current NSCLC patient landscape, as well as treatment and outcomes for mNSCLC patients.

## Patients/material and methods

### Data

This study utilized retrospective data of patients diagnosed at HUS. HUS approved the study (HUS 56/2023), and HUS Data Services collected and pseudoanonymized the data. No ethical approval or informed consent was required (Finnish Act on the Secondary Use of Health and Social Data 552/2019). Findata, the Finnish Social and Health Data Permit Authority, ensured that no identifiable information is published.

### Study cohort

This study included patients with a record of mNSCLC (both de novo and later metastasized) during 2019–2023 who received pharmacological lung cancer treatment. These patients were identified from NSCLC patients residing at HUS region diagnosed during 2017–2022 with a follow-up until death or 31st August 2023, whichever came first. Patients with NSCLC were identified using ICD-10 codes C34.*0-C34.*5 and C34.*9 (for patients with only C34.*0, a contact at pulmonologist and at least two records of C34* ICD-10 codes were required), SNOMED morphology codes in Supplementary Table 1 with lung, pleura, or bronchus as organ, and additionally for the patients with no records of other cancer (ICD-10 code C* other than C34*, C45*, and C76*-80*) SNOMED morphology codes in any organ. NSCLC diagnosis date was set as the earliest record of C34* ICD-10 codes and the SNOMED morphology codes.

Metastatic patients were identified using any records of ICD-10 codes (C78*, C79*), NOMESCO procedure codes AA0* (radiation of brain), and WF049 (radiation of metastasis), TNM M1, Stage IV, SNOMED morphology code M80006, or records of metastasis in patient texts and radiology statements. Index date was set as the earliest record of these.

The study included histological subgroups (SqC and non-SqC) and subgrouping by PD-L1 status (< 1%, 1–49%, 50–100%). Patients with SqC were identified based on SNOMED morphology codes for SqC or ICD-10 code C34.*1, adenocarcinoma using SNOMED morphology codes for adenocarcinoma, or ICD-10 codes C34.*2- C34.*3, and other Non-SqC using other identification codes for NSCLC. For stratifications by PD-L1 status (using the closest PD-L1 record +/- 3 months from the diagnosis), patients with prescription of EGFR, ALK, ROS1, MET, RET, HER2, or NTRK inhibitors or missing PD-L1 were excluded.

### Statistical analysis

Descriptive statistics were mainly used in the data analysis as frequencies (*N*), proportions (%), median, interquartile range (IQR), mean and standard deviation (SD). Overall survival (OS) and time to next treatment (TTNT) were estimated with Kaplan–Meier time to event analysis. Differences in the characteristics across the subgroups were tested using chi-squared (categorical variables), and t-test/Kruskal–Wallis test (continuous variables) using confidence level of 95%. 95% confidence intervals (CIs) were reported for the differences between subgroups in time to event analyses. No adjustment for multiple testing was done. Results with low patient number (1–4 patients) were replaced with ‘< 5’ according to Findata’s instructions for producing anonymous results. Statistical analyses were conducted using R.

### Treatment lines

Treatment lines were constructed using drug administration and prescription data between the index date and end of follow-up. Treatment line was considered to change primarily when the medication(s)/drug(s) changed. If multiple treatments were started within 28 days, it was considered as a combination therapy. A gap of more than 2 years of the same treatment marked a new treatment line. The treatment regimens and lines (first three lines) were visualized using Sankey plots. Treatment regimens were grouped as follows in the Sankey plots: Chemotherapy (ATC codes L01XA*, L01CD*, L01BA04, L01BC05, L01CA04), IO therapy (ATC codes L01FF*, L01FX04, L01FX20) +/- chemotherapy, and TKI therapy (ATC codes L01EB*, L01EC*, L01ED*, L01EH*, L01EX12, L01EX14, L01EX22, L01EX23, L01EX17, L01FX18, L01EX21, L01XX73, L01XX77).

### Treatment outcomes

OS was estimated from start of the first-line (1L) treatment to death (event) or end of study (censoring event), whichever came first. TTNT was estimated from start of the 1L treatment line start to start of the second-line (2L) treatment (event), death (event) or end of study (censoring event), whichever came first. Multivariate Cox proportional hazard model was fitted for the OS from start of 1L including the variables age, sex, histology, and Charlson Comorbidity Index (CCI).

## Results

We identified 646 patients with mNSCLC (Stage IV) who received pharmacological lung cancer treatment with metastasis timepoint between 2019 and 2023 (Table 1). Among these patients, 120 patients had SqC histology, while 526 had non-SqC histology. The majority of non-SqC patients were of adenocarcinoma histology (439 patients, 83%), and 17% (87) were undefined or had other non-SqC histology) (Table 1). Among our mNSCLC patients, those with non-SqC patients were more likely to be diagnosed with metastasis at the initial presentation (83% vs. 70%, *p* = 0.003) and were less likely to be smokers (*p* < 0.001). Additionally, non-SqC patients had fewer significant comorbidities (*p* < 0.001). PD-L1 status was included in our analysis only for those patients for whom it was clinically relevant, namely those not treated with targeted treatment against EGFR, ALK, ROS1, MET, RET, HER2, or NTRK (376 patients, of which 286 Non-SqC and 90 SqC). Brain imaging was performed for 50% of patients. Among those who underwent imaging, brain metastases were found in 49% (135 patients) of non-SqC patients and 35% (17 patients) of SqC patients of those with imaging performed (*p* = 0.01).

**Table 1:** Characteristics of mNSCLC patients at time of NSCLC diagnosis.

Variable	Level	Overall	Non-SqC	SqC	Missing %	p
N		646	526	120		
Age, years, median (IQR)		70 (62–75)	69 (62–75)	71 (64–76)	0	< 0.001
Sex, female, N (%)		319 (49)	281 (53)	38 (32)	0	< 0.001
Histology	Adenocarcinoma	439 (68)	439 (83)	0 (0)	0	< 0.001
	SqC	120 (19)	0 (0)	120 (100)		
	Other Non-SqC	87 (13)	87 (17)	0 (0)		
PD-L1^¶^	< 1%	144 (28)	119 (28)	25 (28)	21	< 0.001
	1–49%	98 (19)	70 (17)	28 (31)		
	50–100%	134 (26)	97 (23)	37 (41)		
Metastatic status^&^	Metastatic at initial diagnosis	518 (80)	434 (83)	84 (70)	0	0.003
	Later progressed to metastatic	128 (20)	92 (17)	36 (30)		
Smoking status^#^	Ex-smoker	282 (49)	223 (48)	59 (53)	11	< 0.001
	Neversmoker	95 (16)	90 (19)	5 (5)		
	Smoker	199 (35)	152 (33)	47 (42)		
ECOG performance status*	0	71 (16)	66 (19)	5 (6)	33	0.021
	1	254 (58)	203 (57)	51 (63)		
	2–4	111 (25)	86 (24)	25 (31)		
CCI (Charlson comorbidity index)	0	285 (44)	247 (47)	38 (32)	0	< 0.001
	1	223 (35)	184 (35)	39 (32)		
	2	93 (14)	63 (12)	30 (25)		
	3	32 (5)	19 (4)	13 (11)		
	4+	13 (2)	13 (2)	0 (0)		
Length of follow-up, months, median (IQR)		30 (15–53)	30 (15–55)	27 (15–52)	0 (0)	0.363

SqC: squamous cell carcinoma; IQR: interquartile range; (ECOG): Eastern Cooperative Oncology Group Performance Status.

*+/- 3months from index, ^&^first record of metastasis between 3 months before or 2 months after diagnosis. ^#^Closest record within one year before diagnosis, or within 90 days after index if no prior records were found. +/-3 months from diagnosis, only patients without targeted treatment against EGFR, ALK or MET inhibitors were included in the PD-L1 analysis.

**Figure 1:**
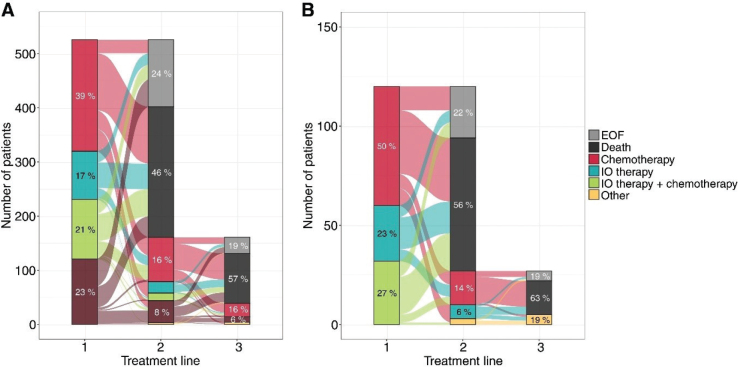
Sankey plot of treatment lines for patients with non-SqC (A) and SqC (B) patients.

Next, we analyzed the treatments stratified by histology (Figure 1). Treatments were categorized into chemotherapy, IO therapy (including PD-L1 and PD-1 inhibitors), IO combined with chemotherapy, and tyrosine kinase inhibitors (TKIs), either alone or in combination with chemotherapy. As expected, TKI therapy was exclusively administered to patients with non-SqC. Throughout the study period, chemotherapy remained the predominant treatment modality. Note, some patients may have received treatment prior to their disease progressing to the metastatic state, meaning the treatments visualized here may not represent their initial lung cancer therapy.

Among those receiving 1L TKI therapy, 53 patients were treated with osimertinib, 22 with afatinib, 22 with alectinib, 14 with gefitinib, and six with crizotinib. These treatments indicate that 89 patients had EGFR mutations (treated with osimertinib, afatinib, or gefitinib), 22 had ALK mutations (treated with alectinib), and six had ALK or ROS1 mutations (treated with crizotinib).

To further explore factors influencing treatment selection, we examined the distribution of first-line treatment types across Charlson Comorbidity Index (CCI) groups (Supplementary Table 2). Patients with higher comorbidity burden (CCI ≥ 3) were more likely to receive chemotherapy alone, while the proportion receiving TKIs decreased with increasing CCI. Proportion of patients receiving IO-based therapies was approximately the same regardless of CCI.

The majority of patients received only one line of treatment (Figure 1). Specifically, 241 patients (46%) with non-SqC and 67 patients (56%) with SqC died before initiating 2L treatment. Meanwhile, 31% with non-SqC and 23% with SqC initiated 2L treatment. The remaining patients were alive at the end of study without records of 2L treatment. For treatment in the overall mNSCLC patients, see Supplementary Figure 1.

**Figure 2:**
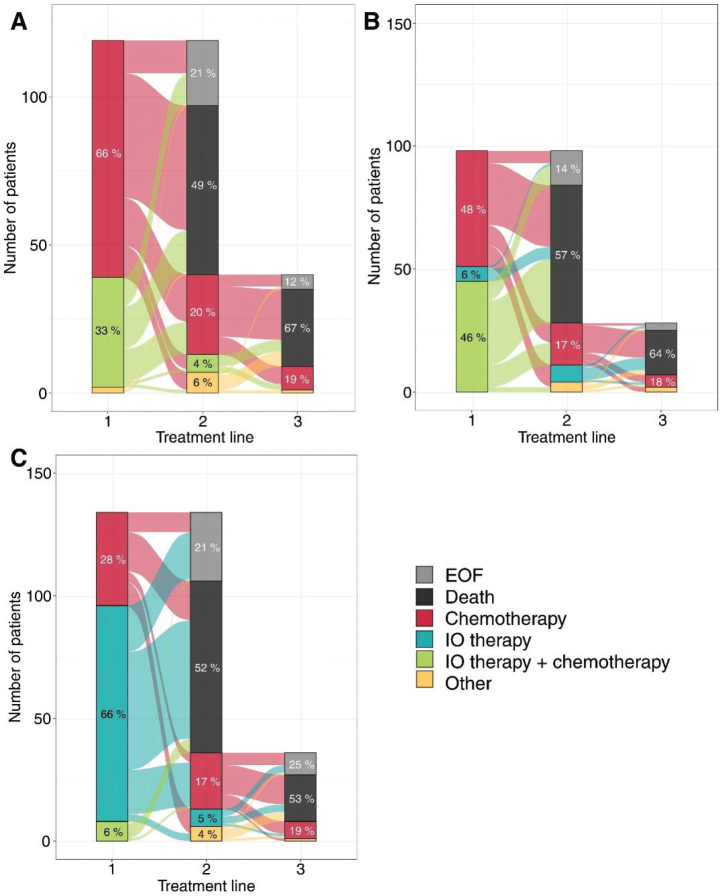
Sankey plot of treatment lines stratified by PD-L1 status. (A) PD-L1 < 1%, (B) PD-L1 1–49% and (C) PD-L1 50–100%.

Next, we analyzed patients stratified by their PD-L1 status (Figure 2 for overall NSCLC, Supplementary Figure 3 for non-SqC). Patients with PD-L1 < 1% were treated in 1L with chemotherapy (66%) and the combination of IO + chemotherapy (33%). Patients with PD-L1 1–49% were treated in 1L with chemotherapy alone (48%), IO therapy either alone (6%), or a combination of IO and chemotherapy (46%). Patients with PD-L1 50–100% were primarily treated in 1L with IO-therapy alone (66%) or in combination with chemotherapy (6%), and less frequently with chemotherapy (28%). The proportion of patients initiating 2L treatment was 30% for PD-L1 < 1%, 29% of PD-L1 1–49%, and 27% of PD-L1 50–100%.

**Figure 3:**
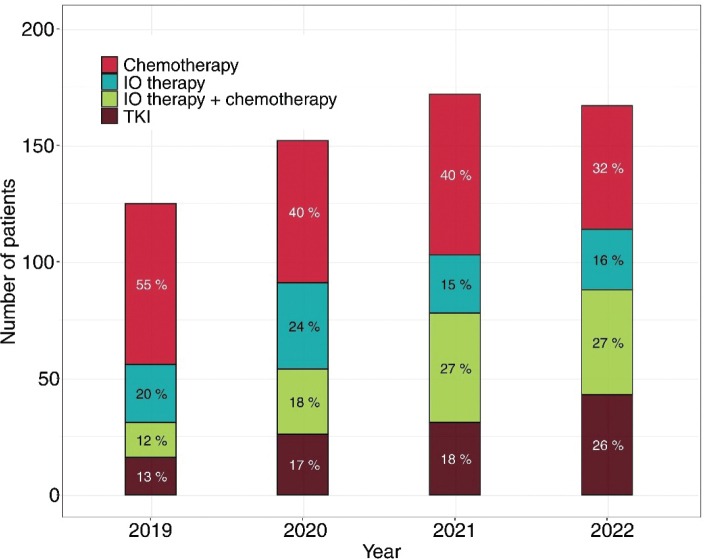
Change in 1L treatment of mNSCLC patients starting 1L treatment during 2019–2022.

When the 1L treatment of all mNSCLC patients was stratified by year of initiating 1L treatment, an increase in the use of IO-treatment, as well as TKI-treatment was seen, with a concurrent decrease in the use of chemotherapy alone (Figure 3). The proportion of patients receiving 1L chemotherapy alone decreased from 55% in 2019 to 32% in 2022, while the proportion of patients receiving 1L IO-therapy (either alone or in combination with chemotherapy) increased from 32% during 2019 to 43% during 2022. In parallel, the use of 1L TKIs doubled from 13% in 2019 to 26% in 2022.

Median overall survival of treated mNSCLC patients was 11 months (95% CI: 10–14, Supplementary Figure 2). When stratified by histology, a trend was observed where non-SqC patients had a longer OS compared to SqC patients. The median OS was 12 months (CI 95% 10–14) for non-SqC and 9 months (CI 95% 7–15) for SqC patients.

**Figure 4:**
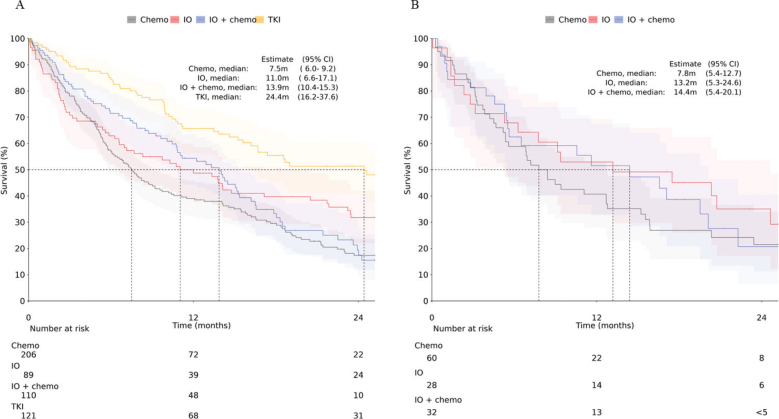
Kaplan–Meier analysis of OS from start of 1L treatment stratified by treatment type (A) non-SqC and (B) SqC. Shaded areas represent 95% CI.

We further stratified OS by type of 1L treatment. Results are presented separately by histological subtype in Figure 4, for the overall mNSCLC cohort in Supplementary Figure 2, and for non-SqC patients by PD-L1 expression subgroups in Supplementary Figure 4 and Supplementary Table 3. The OS for patients treated with chemotherapy was 7.5 months (95% CI: 6.0–9.2) for non-SqC and 7.8 months (95% CI: 5.4–12.7) for SqC. For those treated with IO therapy, the OS was 11.0 months (95% CI: 6.6–17.1) for non-SqC and 13.2 months (95% CI: 5.3–24.6) for SqC. Patients receiving combination therapy had an OS of 13.9 months (95% CI: 10.4–15.3) for non-SqC and 14.4 months (95% CI: 5.4–20.1) for SqC. Non-SqC patients receiving TKI therapy had mOS of 24.4 months (95% CI: 16.2–37.6). These results indicate that the higher OS in non-SqC patients is driven by those receiving TKI therapy.

**Figure 5:**
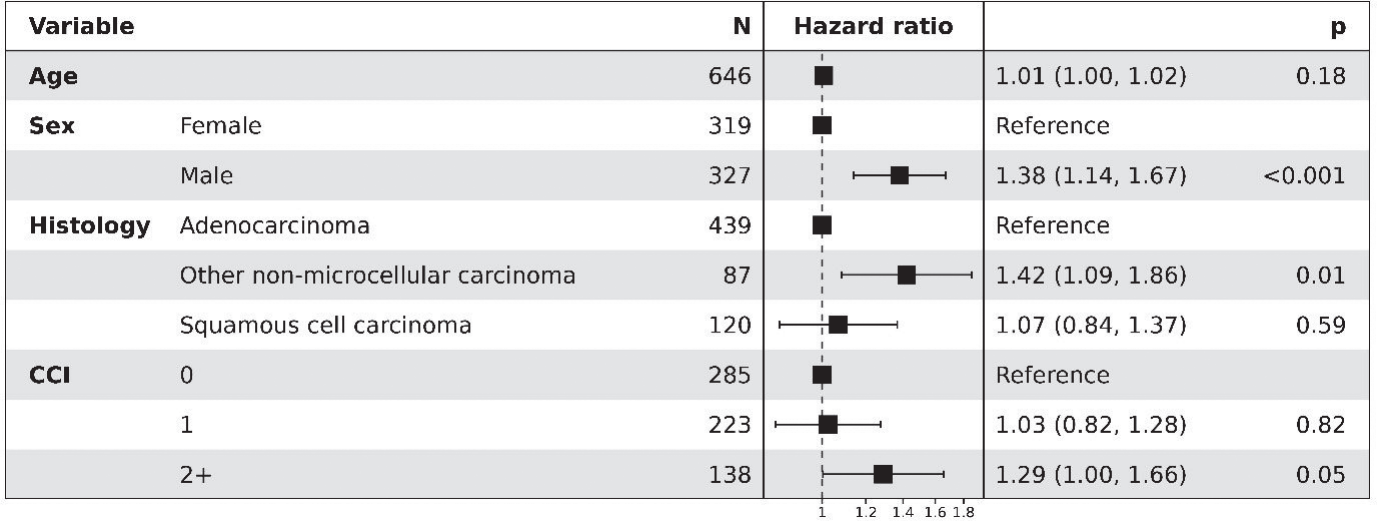
Forest plot of multivariable Cox proportional hazards model for overall survival from start of 1L treatment in patients with mNSCLC.

In multivariable Cox proportional hazards model analysis, male sex was associated with a higher risk of death compared with females (HR 1.38, 95% CI 1.14–1.67; *p* < 0.001). Among histology groups, other NSCLC subtypes had increased risk versus adenocarcinoma (HR 1.42, 95% CI 1.09–1.86; *p* = 0.01), while SqC did not differ significantly (HR 1.07, 95% CI 0.84–1.37, *p* = 0.59). Higher comorbidity burden was associated with worse survival (CCI ≥ 2: HR 1.29, 95% CI 1.00–1.66; *p* = 0.05). Older age was not a significant risk factor of death (HR 1.01 per year; *p* = 0.18) (Figure 5).

**Figure 6:**
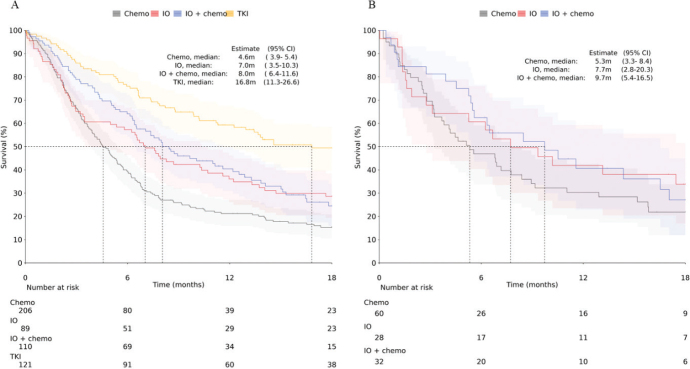
Time to next treatment from the start of 1L to start of 2L for non-SqC (A) and SqC (B) patients. Shaded areas represent 95% CI.

Next, time to next treatment or death (TTNT) was analyzed by histology and treatment type (Figure 6). Here, a similar pattern is seen where chemotherapies have the shortest TTNT (4.6 months for non-SqC, 5.3 months for SqC), whereas TKI treatment has the longest TTNT at 16.8 months (95% CI: 11.3–26.6).

## Discussion and conclusion

This study is the first to provide a RWE of outcomes in the mNSCLC population in Finland, examining the treatment patterns and outcomes of 646 patients during 2019 and 2023. Our findings highlight significant trends and variations in treatment approaches and outcomes, especially when stratified by histology and PD-L1 status.

The pharmacological treatment of patients with mNSCLC has evolved rapidly, positioning mNSCLC at the forefront of both personalized treatment and IO therapy [13]. We restricted the study period to patients diagnosed during 2019–2023, a time when IO therapies were increasingly being adopted. In Finland, 2019 marked the introduction of IO treatments in routine practice, effectively serving as a transition year, and treatment practices continued to evolve thereafter. This evolution is evident in the substantial shift in 1L treatment preferences, with a notable increase in the use of IO and TKI therapies, and a corresponding decrease in the use of chemotherapy alone in 1L and in general. These changes reflect advancements in clinical research and regulatory approvals.

Patients with SqC are not treated with TKIs due to the biological characteristics and lack of indication. Because patients with SqC and a smoking history rarely have tumors harboring actionable mutations, they are generally not candidates for TKI treatment, and according to international guidelines, routine genetic testing is recommended only for SqC cases in never-smokers or light former smokers. Treatment decisions, including the use of immuno-oncology (IO) therapy guided by PD-L1 status, aligned with the treatment guidelines during the study period [14]. Differences in OS were observed among treatment modalities, with 1L chemotherapy patients having the shortest OS, and TKI treated patients having the longest. These OS outcomes are consistent with international studies [15–17] and are likely influenced by variations in baseline characteristics and disease biology. Registrational trials reported median OS of 22 months for chemo-immunotherapy in non-SqC NSCLC [18] and 17 months in squamous [19], compared with ~10–12 months for chemotherapy alone, while EGFR TKIs achieved 38.6 months in FLAURA [20]. Recent Nordic real-world data, including Swedish [21] and Norwegian [22] studies, show similar patterns, with the Swedish study reporting medians of 15.2 months (non-SqC) and 12.9 months (SqC) for IO-therapy alone, and the Norwegian study demonstrating comparable improvement after the introduction of IO-therapy, broadly consistent with our findings. Differences between trial and real-world outcomes are expected, as RCTs enroll selected patients under controlled conditions, whereas real-world cohorts reflect broader clinical practice, making such data important for understanding outcomes in routine care.

This study leverages the comprehensive Finnish hospital data lake, which allow the integration of deep clinical data. The universal healthcare system in Finland, characterized by equal access provides a robust foundation for real-world studies. Our cohort, drawn from the largest provider of specialized care in Finland covering about 30% of the Finnish population, ensures broad representativeness of the national NSCLC patient population. Due to standardized treatment protocols across Finland, these results are likely applicable to the entire country [1]. Limitations of this study include the retrospective registry design, which leads to issues such as missing data, particularly regarding Eastern Cooperative Oncology Group Performance Status (ECOG) performance scores, and coverage of only one area with a limited number of patients.

In conclusion, our study provides valuable insights into the evolving treatment landscape of mNSCLC in Finland. The increasing use of IO and TKI therapies reflects advancements in personalized medicine, and our findings underscore the importance of molecular profiling in guiding treatment decisions.

## Supplementary Material



## Data Availability

Since this study is based on the secondary use of healthcare registry data, these data cannot be shared openly.
