# Supplementary material for: Metastatic NSCLC patients in the real world in Finland
Source: Acta Oncol. 2025 Dec 15;64:44254. doi: 10.2340/1651-226X.2025.44254 (PMC12717630; doi:10.2340/1651-226X.2025.44254)
Supplement: Supplementary file 1 [file AO-64-44254-s1.pdf]

Supplementary material has been published as submitted. It has not been copyedited, or typeset by Acta Oncologica

SUPPLEMENTAL DATA:

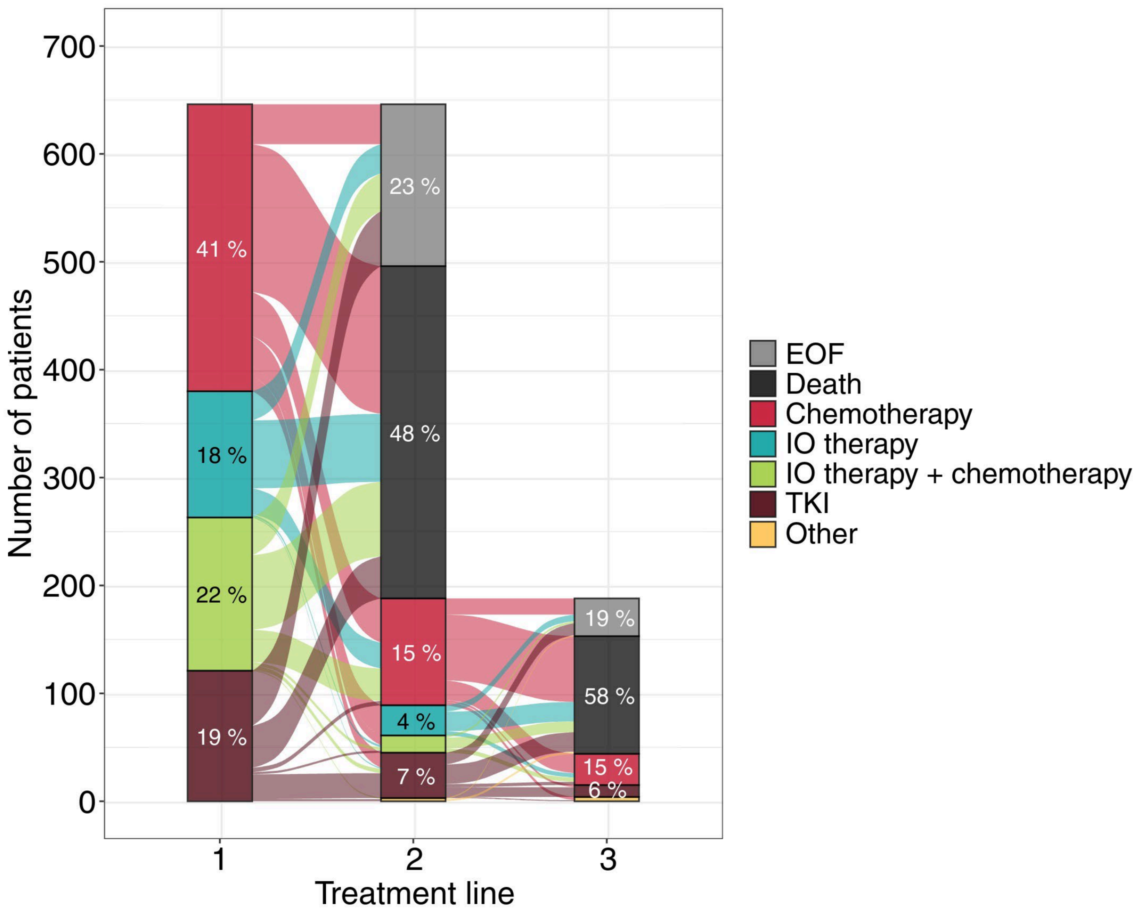

Supplementary Figure 1. *Sankey plot of treatment lines for overall mNSCLC cohort.*

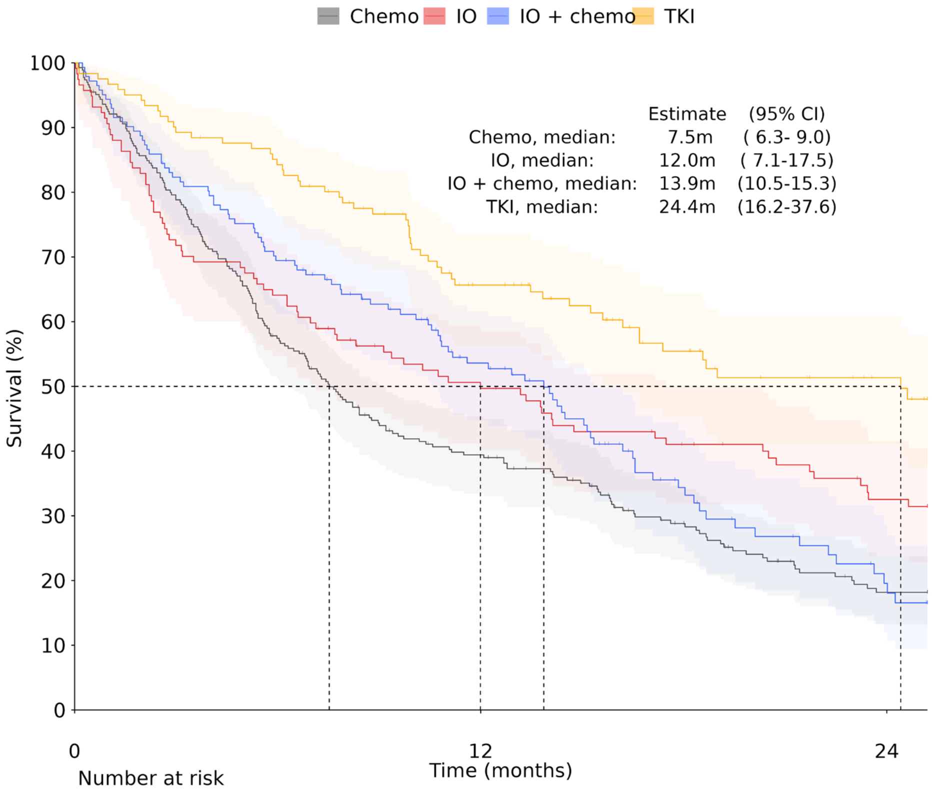

Number at risk

	0	12	24
Chemo	266	94	30
IO	117	53	30
IO + chemo	142	61	13
TKI	121	68	31

Supplementary Figure 2. *Overall survival from start of 1L stratified by treatment. Shaded areas represent 95% CI.*

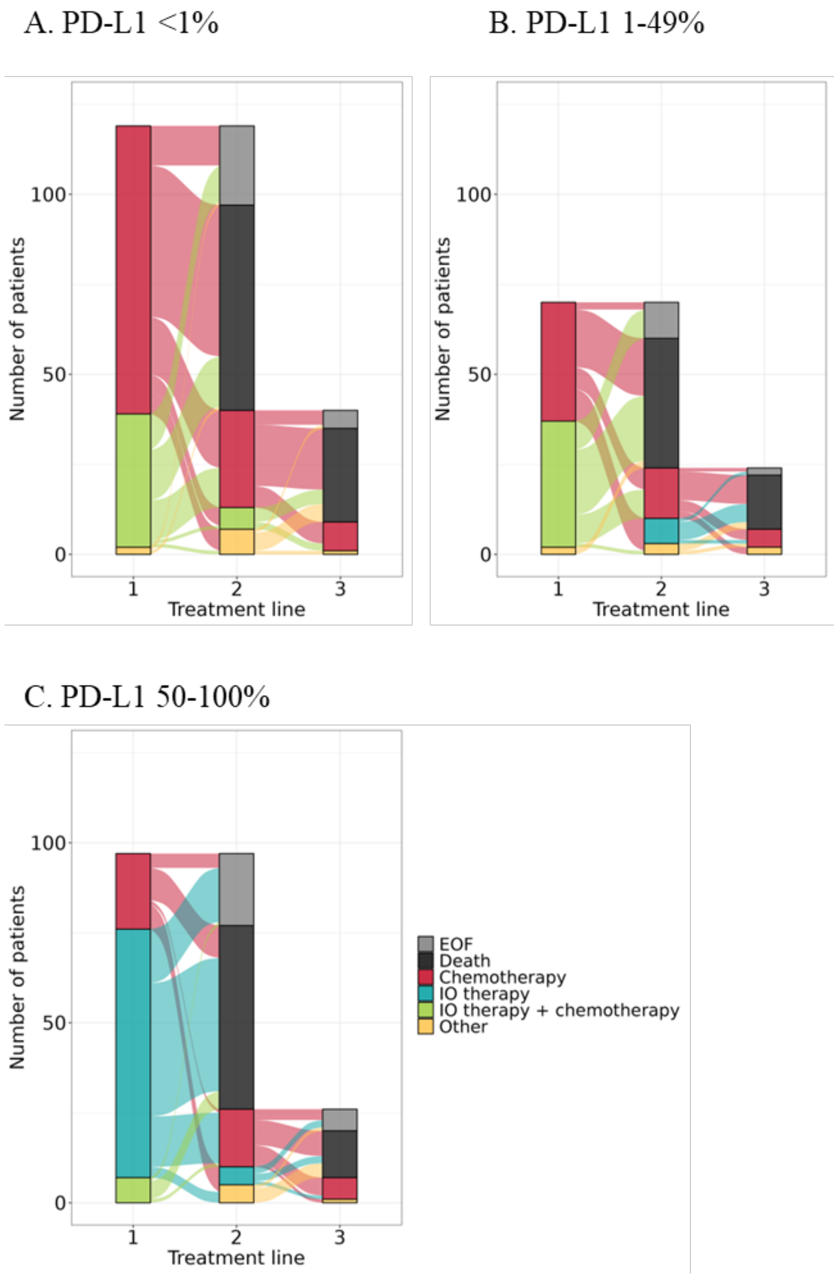

Supplementary Figure 3. *Sankey plot of treatment lines for non-SqC patients stratified by PD-L1 status. A) PD-L1 <1%, B) PD-L1 1-49% and C) PD-L1 50-100%.*

A. PD-L1 <1%

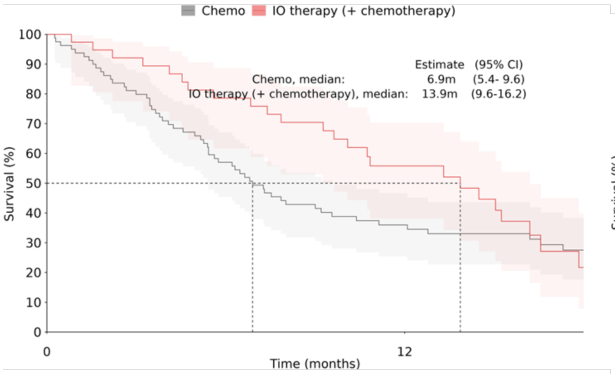

B. PD-L1 1-49%

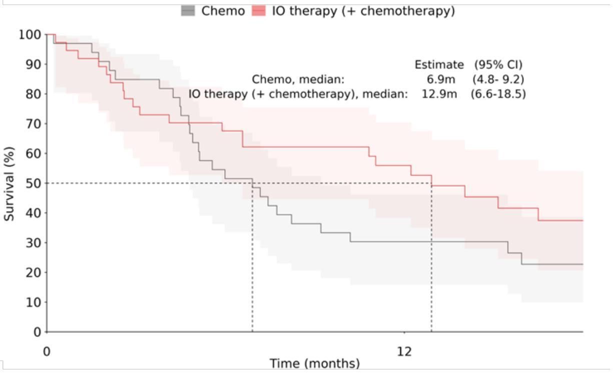

C. PD-L1 50-100%

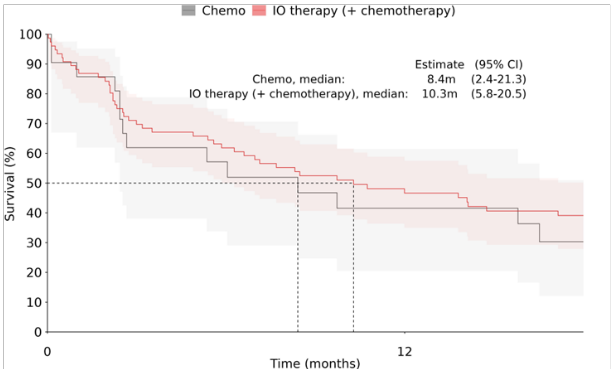

Supplementary Figure 4. *Overall survival from start of 1L stratified by 1L treatment (chemotherapy alone versus immunotherapy or chemo-immunotherapy), for non-SqC patients stratified by PD-L1 expression level:*

(A) PD-L1 < 1%, (B) PD-L1 1–49%, and (C) PD-L1 ≥ 50%. Median OS (months) with 95% CI is shown for each group

Supplementary Table 1. *SNOMED morphology codes for NSCLC identification and histology definition.*

Histological subgroup	Histology	SNOMED morphology codes
Squamous cell carcinoma (SqC)	Squamous cell carcinoma	M80703
		M80706*
Non-squamous cell (Non-SqC)	Adenocarcinoma	M81403
		M81406*
	Other Non-SqC	M80123
		M85603
		M80103
		M80106*

if no records of other cancer (ICD-10 code C other than C34*, C45*, and C76*-80*)

Supplementary Table 2. *Distribution of first-line treatment types across CCI groups.*

CCI group	Treatment group	N	%
**0**	**Chemotherapy**	99	35
	**IO-therapy**	44	15
	**IO therapy + chemotherapy**	68	24
	**TKI**	74	26
**1**	**Chemotherapy**	103	46
	**IO-therapy**	42	19
	**IO therapy + chemotherapy**	46	21
	**TKI**	32	14
**2**	**Chemotherapy**	41	44
	**IO-therapy**	22	24
	**IO therapy + chemotherapy**	19	20
	**TKI**	11	12
**3+**	**Chemotherapy**	23	51
	**IO-therapy**	9	20
	**IO therapy + chemotherapy**	9	20
	**TKI**	4	9

Supplementary Table 3. *Outcomes from start of 1L treatment for non-SqC patients by PD-L1 expression.*

**PD-L1 expression**	**Treatment group**	**TTNT, months, median (95% CI)**	**OS, months, median (95% CI)**
**<1%**	**Overall**	6.1 (4.9 - 6.5)	9.2 (6.8 -12.1)
	**Chemotherapy**	5.2 (3.8 - 6.2)	6.9 (5.4 - 9.6)
	**IO therapy +/- chemotherapy**	7.4 (6.1 - 13.8)	13.9 (9.6 - 16.2)
**1-49%**	**Overall**	6.5 (4.4 - 9.7)	8.7 (5.9 - 15.1)
	**Chemotherapy**	4.5 (3.0 - 5.9)	6.9 (4.8 - 9.2)
	**IO therapy +/- chemotherapy**	11.6 (6.5 - 15.1)	12.9 (6.6 - 18.5)
**50-100%**	**Overall**	5.7 (3.0 -7.9)	9.7 (6.0 -15.8)
	**Chemotherapy**	2.8 (1.5 - 5.4)	8.4 (2.4 - 21.3)
	**IO therapy +/- chemotherapy**	7.3 (3.2 - 10.3)	10.3 (5.8 - 20.5)
